# Other (Non-CNS/Testicular) Extramedullary Localizations of Childhood Relapsed Acute Lymphoblastic Leukemia and Lymphoblastic Lymphoma—A Report from the ALL-REZ Study Group

**DOI:** 10.3390/jcm10225292

**Published:** 2021-11-14

**Authors:** Andrej Lissat, Claudia van Schewick, Ingo G. Steffen, Ayumu Arakawa, Jean-Pierre Bourquin, Birgit Burkhardt, Guenter Henze, Georg Mann, Christina Peters, Lucie Sramkova, Cornelia Eckert, Arend von Stackelberg, Christiane Chen-Santel

**Affiliations:** 1 Department of Pediatric Hematology and Oncology, Charité-Universitätsmedizin Berlin, Augstenburger Platz 1, 13353 Berlin, Germany; Claudia.schewick@charite.de (C.v.S.); Ingo.steffen@charite.de (I.G.S.); aarakawa@ncc.go.jp (A.A.); guenter.henze@charite.de (G.H.); cornelia.eckert@charite.de (C.E.); arend.stackelberg@charite.de (A.v.S.); christiane.chen-santel@charite.de (C.C.-S.); 2 Eleonoren-Foundation, Pediatric Hematology Oncology Department, University Children’s Hospital Zürich, Steinwiesstraße 75, CH-8032 Zürich, Switzerland; jean-pierre.bourquin@kispi.uzh.ch; 3 Department of Pediatric Hematology and Oncology, Universitätsklinikum Münster, Albert-Schweitzer-Campus 1, Gebäude A1, 48149 Münster, Germany; birgit.burkhardt@ukmuenster.de; 4 Department of Pediatric Hematology and Oncology, St. Anna Children’s Hospital, Kinderspitalgasse 6, A-1090 Vienna, Austria; georg.mann@stanna.at (G.M.); christina.peters@stanna.at (C.P.); 5 Charles University 2nd Faculty of Medicine and UH Motol V Uvalu 84, Prague 5 15006, Czech Republic; Lucie.Sramkova@fnmotol.cz

**Keywords:** other extramedullary relapse, pediatric, lymphoblastic leukemia

## Abstract

Children with other extramedullary relapse of acute lymphoblastic leukemia are currently poorly characterized. We aim to assess the prevalence and the clinical, therapeutic and prognostic features of extramedullary localizations other than central nervous system or testis in children with relapse of acute lymphoblastic leukemia (ALL) and lymphoblastic lymphoma (LBL) treated on a relapsed ALL protocol. Patients and Methods: Patients with relapse of ALL and LBL, treated according to the multicentric ALL-REZ BFM trials between 1983 and 2015, were analyzed for other extramedullary relapse (OEMR) of the disease regarding clinical features, treatment and outcome. Local treatment/irradiation has been recommended on an individual basis and performed only in a minority of patients. Results: A total of 132 out of 2323 (5.6%) patients with ALL relapse presented with an OEMR (combined bone marrow relapse *n* = 78; isolated extramedullary relapse *n* = 54). Compared to the non-OEMR group, patients with OEMR had a higher rate of T-immunophenotype (*p* < 0.001), a higher rate of LBL (*p* < 0.001) and a significantly different distribution of time to relapse, i.e., more very early and late relapses compared to the non-OEMR group (*p* = 0.01). Ten-year probabilities of event-free survival (pEFS) and overall survival (pOS) in non-OEMR vs. OEMR were 0.38 ± 0.01 and 0.32 ± 0.04 (*p* = 0.0204) vs. 0.45 ± 0.01 and 0.37 ± 0.04 (*p* = 0.0112), respectively. OEMRs have been classified into five subgroups according to the main affected compartment: lymphatic organs (*n* = 32, 10y-pEFS 0.50 ± 0.09), mediastinum (*n* = 35, 10y-pEFS 0.11 ± 0.05), bone (*n* = 12, 0.17 ± 0.11), skin and glands (*n* = 21, 0.32 ± 0.11) and other localizations (*n* = 32, 0.41 ± 0.09). Patients with OEMR and T-lineage ALL/LBL showed a significantly worse 10y-pEFS (0.15 ± 0.04) than those with B-Precursor-ALL (0.49 ± 0.06, *p* < 0.001). Stratified into standard risk (SR) and high risk (HR) groups, pEFS and pOS of OEMR subgroups were in the expected range whereas the mediastinal subgroup had a significantly worse outcome. Subsequent relapses involved more frequently the bone marrow (58.4%) than isolated extramedullary compartments (41.7%). In multivariate Cox regression, OEMR confers an independent prognostic factor for inferior pEFS and pOS. Conclusion: OEMR is adversely related to prognosis. However, the established risk classification can be applied for all subgroups except mediastinal relapses requiring treatment intensification. Generally, isolated OEMR of T-cell-origin needs an intensified treatment including allogeneic stem cell transplantation (HSCT) as a curative approach independent from time to relapse. Local therapy such as surgery and irradiation may be of benefit in selected cases. The indication needs to be clarified in further investigations.

## 1. Introduction

Relapses in the central nervous system (CNS) and testis account for 87% of all extramedullary relapses in childhood ALL. These sites are considered sanctuary sites inhibiting the efficacy of systemic chemotherapy. In addition, interaction and biology of leukemic blast (sub)populations with the specific organ microenvironment can induce quiescence, prevent apoptosis and lead to treatment failure and relapse in these sanctuary sites (review in [1]). In CNS and testicular relapse, a specific local treatment (irradiation, intrathecal injections, orchiectomy) combined with systemic chemotherapy and in high-risk patients additional allogeneic hematopoietic stem cell transplantation (HSCT) is needed to induce long-term remission in patients suffering from relapse at these particular sites [2,3].

In contrast, ALL relapses in extramedullary compartments other than CNS or testis are poorly characterized and not addressed with specific treatment recommendations in current treatment protocols. Due to the relative rarity of these so-called other extramedullary relapses (OEMRs) and the large heterogeneity of organs involved, primarily case reports on single-center experience with unusual extramedullary localizations have been published so far [4,5,6,7,8,9,10,11,12,13,14,15,16,17,18,19,20,21,22,23]. Gunes and colleagues described other extramedullary relapses in 6 out of 51 adult and adolescent ALL patients following HSCT. OS after HSCT in all OEMR patients has not been significantly different compared to isolated BM relapses. The small patient number of OEMR precluded any statistical analysis [24]. Only a few analyses describing a cohort of more than 10 patients have been reported so far [25,26].

The lack of comprehensive clinical data and strong evidence impairs stratification of affected pediatric patients to systemic and local treatment. Clinical trial protocols of subsequent ALL—Relapse Berlin/Frankfurt/Muenster (ALL-REZ BFM) trials for childhood relapsed ALL recommend systemic chemotherapy for these patients without addition of local treatment. The latter is recommended only in case of tumor mass persistence after induction and consolidation verified by pathologic review and in sanctuary sites such as the eye. The reason for the current approach is based on the assumption that in OEMR no sanctuary mechanism such as the blood–brain barrier would prevent the efficacy of systemically administered chemotherapeutic drugs. However, it remains unclear whether risk group allocation, treatment intensity and strategy for local therapy in OEMR ALL are adequate for these patients and subgroups. 

To improve treatment stratification by analysis of relapse patterns, response and survival in pediatric OEMR ALL patients, we summarized data of the entire cohort of patients enrolled into five consecutive ALL-REZ BFM trials conducted between 1983 and 2015. Herewith, we present the largest analysis of a pediatric cohort of OEMR ALL published so far, enabling us to better characterize its clinical and prognostic features as well as its therapeutic needs. 

## 2. Materials and Methods

### 2.1. Patients

Between June 1983 and March 2015, 2323 children and adolescents with diagnosis of first relapsed ALL and LBL were enrolled into the randomized multicenter ALL—Relapse trials as well as registries of the ALL-REZ BFM study group. Of these, 132 were diagnosed with extramedullary relapse in compartments other than CNS or testis. Seventy-eight patients of this cohort presented with combined extramedullary and bone marrow relapse, and 54 were diagnosed with isolated extramedullary relapse. Written informed consent was obtained from patients and/or their guardians. Protocols of the trials ALL-REZ BFM 83, 85, 87, 96 and 2002 were approved by the institutional ethics committees of the participating institutions. The ALL-REZ BFM 2002 trial has been registered in the International Clinical Trials Registry Platform of the WHO (NCT00114348).

### 2.2. Definitions 

Isolated extramedullary relapse has been defined as clinically overt relapse in an extramedullary compartment and less than 5% leukemic lymphoblasts in the bone marrow (BM). Combined extramedullary and BM relapse has been defined as extramedullary involvement and ≥5% BM blast infiltration. OEMR was diagnosed by biopsy in the majority of patients and by ultrasound, computer tomography, magnetic resonance imaging or scintigraphy. 

Lymph node involvement was diagnosed in case of lymphatic mass beyond the usual lymphadenopathy with lymph node diameter > 2 cm assessed at the discretion of the treating PI. Biopsy was recommended in all isolated lymphatic organ relapse patients to secure diagnosis. 

Time point of relapse has been defined as follows: very early, relapse within 18 months after diagnosis; early, relapse later than 18 months after diagnosis but less than 6 months after cessation of front-line treatment; late, relapse 6 months after end of front-line treatment. 

Routine immunophenotyping and analyses for chromosomal translocations were performed as described elsewhere [27,28].

Risk stratification has been based on standard of care related to current definitions within the IntReALL protocol and ALL REZ clinical trials: 

SR included: early isolated extramedullary (IEM) and combined bone marrow (CBM) BCP-ALL relapses; early IEM T-ALL relapses; late IEM, CBM and isolated bone marrow (IBM) BCP-ALL relapses; and late IEM T-ALL relapses. 

HR included: all very early BCP- and T-ALL relapses, early IBM BCP-ALL and early CBM and IBM T-ALL relapses and late CBM and IBM T-ALL relapses. 

S1 group: late IEM BCP- and T-ALL relapses, according to SR.

S2 group: early IEM and CBM and late IBM BCP-ALL relapses and early IEM T-ALL relapse, according to SR.

S3 group: early IBM BCP-ALL relapse, according to HR.

S4 group: all very early BCP- and T-ALL relapses excluding S1 and S2, according to HR.

Definition of nonresponse: in patients with BM involvement, absence of complete morphological remission (CMR) (<5% lymphoblasts) at the fifth therapy element (e.g., ALL-REZ BFM 2002: at day 29 Prot. II-IDA). 

Likewise, in IEM relapse including OEMR no evidence of local disease was considered as complete remission and evidence of disease—in OEMR proven by biopsy—was assessed as nonresponse.

### 2.3. Treatment

All patients received either alternating courses of systemic and intrathecal chemotherapy (R1 and R2 blocks, since protocol ALL-REZ BFM 95 all groups started with F1- and F2-induction blocks) or continuous chemotherapy with lower dosage, but for a longer time period (protocol II-IDA) according to the ALL-REZ BFM protocols 85, 87 [29], 90 [30], 96 [31] and 2002 [32]. Cranial irradiation of 12 gray (Gy) was administered to all patients with CNS involvement. Treatment for OEMR did not differ from the approach for systemic relapse as has been published before [33]. As mentioned above, irradiation has not been recommended as standard of care in OEMR. Only 17 patients received additional local irradiation for the extramedullary compartment with the application of doses from 10 to 30 Gy based on individual choice and recommendation independent of response. Allogeneic HSCT was indicated in patients stratified into the high-risk group (S3/S4) with very early bone marrow involving relapse and since the trial ALL-REZ BFM 2002 in patients with minimal residual disease (MRD) poor response after induction chemotherapy or other high-risk features according to the ALL SZT-BFM 2003 trial and the international FORUM study which was initiated in 2012 [34]. HLA compatible siblings and if available unrelated donors have been considered as suitable stem-cell donors. In recent years, HLA mismatched family donors have also been used in high-risk patients. Conditioning regimen for children above 2 or recently above 4 years included total body irradiation with 12 Gy in the majority of the patients. 

### 2.4. Statistical Methods

The association of categorical variables was analyzed using Pearson’s chi-squared test and Fisher’s exact test (*n* ≤ 5/cell). EFS time was calculated from the date of relapse diagnosis to the date of an event (i.e., second relapse, therapy-related death and secondary malignancy) or the date of last follow-up. In case of nonresponse or death over the course of induction therapy, EFS time was set to zero. The probability of event-free survival (pEFS) and the probability of overall survival (pOS) were estimated by the Kaplan–Meier life-table method [34], and differences between groups were assessed by the log-rank test. The effect of prognostic factors on EFS and OS was analyzed using univariate and multivariate Cox proportional hazard regression model and the corresponding hazard ratios and their 95% confidence intervals (CIs). Akaike information criterion (AIC) minimization method was used to optimize the multivariate Cox regression model. All tests were two-sided and the significance level was set to *p* < 0.05. The software R 4.0.3 (R Foundation for Statistical Computing, Vienna, Austria), SAS 9.4 (SAS Institute, Cary, NC, USA) and SPSS 27.0 (IBM SPSS Statistics, Ehningen, Germany) were used for statistical analysis.

## 3. Results

### 3.1. Clinical Presentation of OEMR Differs Significantly from Non-OEMR Patients

One hundred thirty-two children with OEMR manifestations of ALL and LBL were included in the analysis. Involvement of 17 distinct extramedullary sites has been observed. The most frequent sites for OEMR were mediastinum (*n* = 35), lymph nodes (*n* = 32), skin (*n* = 14) and bone (*n* = 12). Localizations in organs such as the kidney (*n* = 9), eye/orbit (*n* = 4) or liver (*n* = 3) were rare (for a list of all sites and their distribution see Table 1 and Suppl. Figure S1). 

We grouped OEMRs into five categories according to the main extramedullary compartment involved: “mediastinum” (*n* = 35), “lymphatic organs” (*n* = 32), a group named “other compartment” including all patients with localizations that did not fit in one of the other four groups (*n* = 32), “skin and glands” (*n* = 21) and “bone” (*n* = 12). In the case of more than one OEMR localization in one patient, only the main site as reported by the treating PI was considered for statistical analysis between the subgroups. 

Compared with the entire non-OEMR cohort of 2191 first relapsed ALL patients, patients with OEMR presented more frequently a T-immunophenotype leukemia (OEMR 50.8%, *n* = 67, versus non-OEMR 11.0%, *n* = 242; *p* ≤ 0.001; for complete analysis see Table 2). In addition, more patients in the OEMR group showed very early or late relapses, (31.8% and 49.2%, respectively) compared to the non-OEMR group (23.9% and 45.7%, respectively, *p* = 0.01). Significantly more patients in the OEMR group have been treated on T-LBL protocols during first-line therapy, 15.2% vs. 1.9% in the non-OEMR group (*p* < 0.001), most likely representing former T-LBL patients. Gender, age and the rate of HSCT in consolidation did not differ significantly in OEMR vs. non-OEMR subgroups. 

Molecular data on specific translocations (*BCR-ABL1*, *MLL-AF4* and *ETV6-RUNX1)* were available in 32.7% (*n* = 43) of OEMR patients. This lack of data was mainly caused by the long observation period covering early periods when genetic diagnostics had not been routinely performed, as well as the difficulty of performing genetic analyses in extramedullary material in general (Supplementary Table S1). One OEMR patient had evidence of a leukemia with TEL-AML fusion, and another patient was diagnosed with BCR-ABL fusion. The remaining 41 patients did not show any of the investigated genetic aberrations currently applied for risk stratification. Genetic characteristics were not recorded or reported in 67% of OEMR patients. This precludes any statement on the correlation of underlying genetic features with risk of OEMR or definition of new biomarkers which need to be established prospectively. 

### 3.2. OEMR Subgroups Demonstrate Distinct Relapse Phenotypes 

To improve treatment stratification, we analyzed high-risk patterns within OEMR subgroups (Table 2). T-immunophenotype was predominant in the mediastinal mass group and more frequent in the lymphatic organs group (94.3% (*n* = 33) and 56.2% (*n* = 18), respectively; *p* < 0.001). In the “skin/gland” and “other” OEMR subgroups, T-ALL subtype was diagnosed in a minority of patients (28.6%, *n* = 6; 28.1%, *n* = 9). In the “bone” OEMR subgroup, relapsed BCP-ALL subtype was far more frequent than T-ALL (91.7%, *n* = 11, vs. 8.3%, *n* = 1, respectively). In addition to phenotype, time to relapse differed significantly in the five OEMR subgroups (*p* = 0.04). Mediastinal and bone relapses occur more frequently in the very early (43%, 42%) and early (31%, 17%) relapse groups, whereas the subgroups “other”, “skin/gland” and “lymphatic organs” occur predominantly as late relapses (69%, 62%, 50%; Table 2).

Since T-LBL and pB-LBL patients have been included in and treated according to clinical trial protocols for relapsed ALL in the past, our analysis included 43 patients who suffered from T-LBL and 19 patients who suffered from pB-LBL as primary disease and were treated according to NHL-BFM first-line protocols. Out of these 62 patients, 20 (32%) relapsed as lymphoblastic leukemia/lymphoma including an OEMR site (16 T-LBL and 4 pB-LBL). These 20 patients comprise 15% (20/132) of the OEMR cohort analyzed and are thus overrepresented as compared to the non-OEMR cohort (*p* < 0.001; Table 2). As expected, the “mediastinal” and “lymph node” OEMR subgroups comprise the majority—17—of these 20 LBL patients. Within these two subgroups, former LBL patients comprise 26% and 22% of patients, respectively. The vast majority of patients included in the OEMR analysis had been treated according to a first-line ALL protocol, i.e., 112 out of 132 patients. Sex, age and therapy did not show a significantly different distribution within the five OEMR cohorts.

### 3.3. OEMR Shows a Distinct Event Pattern Compared to Non-OEMR

Events are summarized in Table 3a. Relapse rate and complete continuous remission (CCR) did not differ significantly in OEMR vs. non-OEMR patients (Table 3a). We found significantly more deaths in induction and nonresponding patients in the OEMR group (non-OEMR vs. OEMR 3.7% vs. 8.3% (*p* = 0.02) and 9.8% vs. 15.9% (*p* = 0.03), respectively), which might be attributed to different risk patterns in both groups. Mediastinal and bone relapses were associated with “nonresponse to treatment/progressive disease” and “death in induction” (43% in the “mediastinal” and 42% in the “bone” vs. 14% in the remaining three OEMR subgroups). Fewer patients within the “mediastinal” and “bone” subgroups compared to the remaining three OEMR subgroups stayed in CCR, 11% and 17% vs. 42%, respectively. In contrast to that, in the group “lymphatic organs”, more patients remained in CCR than any other subgroup (50%, *n* = 16). 

To understand the relapse pattern of OEMR subgroups in detail and to improve recommendation on local therapy, we took a closer look at the site of the subsequent relapse (Table 3b,c). Out of 132 patients in the OEMR cohort, 48 experienced a subsequent relapse. Of these 48 patients, 28 (58%) relapsed as combined bone marrow (*n* = 8) or isolated extramedullary (*n* = 20) relapse, which differs significantly from non-OEMR patients, where only 23.5% relapsed as CBM or IEM (*p* < 0.001; Table 3b). Forty-two percent of observed subsequent relapses were isolated bone marrow relapses (20 patients). Within the “other” OEMR cohort, subsequent relapses occurred predominantly as isolated OEMR (62%; Table 3b). Only 9 out of 28 subsequent extramedullary relapses involved the initial relapse site. The majority of these relapses involved other EM sites including CNS and testis. Within the “other” OEMR subgroup, 3 out of 9 subsequent relapses involved the initial site (Table 3c). 

As a consequence, we focused on the value of additional local irradiation on outcome in OEMR patients (Supplementary Table S2). In general, the ALL-REZ BFM protocols combine systemic and intrathecal chemotherapy as well as radiation in certain defined subgroups. However, since most of the OEM sites are not considered sanctuary sites, local radiation has not been recommended as standard of care. In general, out of 128 OEMR patients, on whom information on radiation was available, only a minority of 17 patients (13%) received local irradiation (*n* = 15) or local radiation combined with TBI (*n* = 2) whereas the majority did not (Supplementary Table S2). As an exception, relapses within the eye have been considered as specific local risk being potentially protected from chemotherapeutic agents by a blood–retina barrier [35]. Three out of four patients with ocular relapses received irradiation of the eye. No subsequent relapses were reported in these patients. Furthermore, 10 patients with mediastinal relapse (one patient in combination with TBI) and 5 patients belonging to the “other” subgroup (one patient in combination with TBI) underwent local radiotherapy. Final conclusions on the indication for specific local radiation therapy cannot be drawn. This needs to be addressed in further preferably prospective analyses. However, we would continue recommending local irradiation of sanctuary sites such as relapses within the eye.

Risk stratification and indication to undergo allogeneic HSCT in OEMR patients have been recommended based on established algorithms for all relapsed patients. However, detailed analysis revealed subtle differences in HSCT rate in non-OEMR vs. OEMR patients (Table 4a,b). Thirty-two patients (24%) with OEMR underwent an allogeneic HSCT. Nine of these belonged to the HR group (S4), and 23 belonged to the S1 and S2 group (SR), who are transplanted based on MRD response. Unfortunately, data on MRD response in the OEMR group were not available in the majority of patients, precluding a deeper insight into the indication of SCT and meeting criteria to perform the latter. Compared to non-OEMR patients, more patients in OEMR S1 underwent allogeneic HSCT (0% vs. 16.7%, respectively) and fewer patients in OEMR S4 underwent HSCT (32.0% vs. 20.9%, respectively). The latter could be partly attributed to overrepresentation of T-ALL and very early relapsed patients in the OEMR cohort, which both are associated with nonresponse to induction and refractoriness precluding HSCT. 

Outcome following allogeneic HSCT in non-OEMR and OEMR patients did not show substantial differences. Due to selection biases and time dependency of HSCT, we did not perform statistical analysis on outcome after HSCT vs. chemotherapy alone in non-OEMR vs. OEMR patients (Table 4b). In general, OEMR patients who underwent HSCT experienced a considerable CCR rate of 44% (non-OEMR 53%). OEMR patients treated with chemotherapy alone experienced a CCR rate of 38% (non-OEMR 37%). The rate of subsequent relapses in the OEMR HSCT group was 38% (non-OEMR 28.7%) compared to 55% (non-OEMR 58%) in patients treated with chemotherapy only. The death-in-remission rate in the OEMR SCT group was 13% (non-OEMR 5%) (Table 4b). Based on these data, an HSCT stratification algorithm including HLA-mismatched donors for OEMR cannot be established, and recommendation for HSCT should be based on contemporary risk criteria.

### 3.4. OEMR Confers an Independent Risk Factor for Decreased Survival

**Figure 1:**
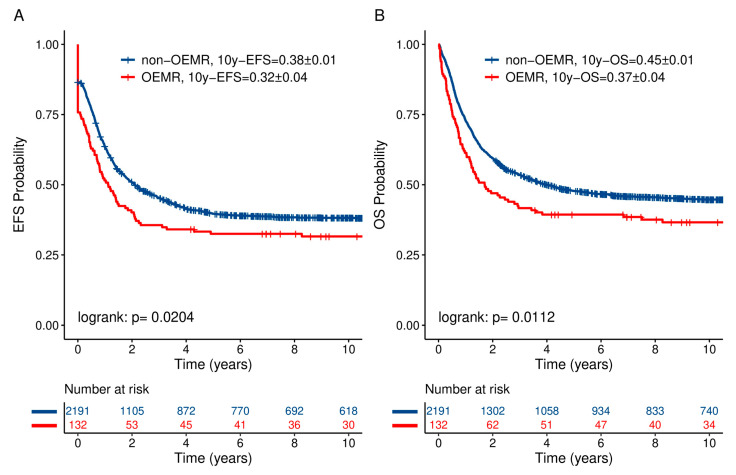
Ten-year pEFS (**A**) and pOS (**B**) of relapsed ALL patients differ significantly in OEMR vs. non-OEMR patients. The graphs have been calculated based on Kaplan–Meier analysis. *p* < 0.05.

**Figure 2:**
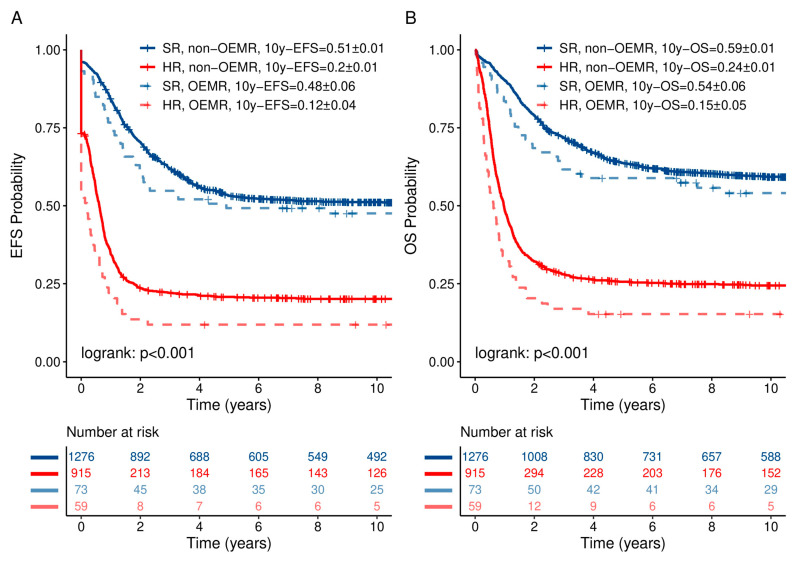
Ten-year pEFS (**A**) and pOS (**B**) of SR vs. HR patients differ significantly in OEMR and non-OEMR patients. The graphs have been calculated based on Kaplan–Meier analysis. Pairwise log-rank test applied in subgroup analysis. *p* < 0.001.

The probability of 10-year event-free survival (10y-pEFS) and the probability of 10-year overall survival (10y-pOS) in comparison to the non-OEMR ALL cohort are shown in Figure 1 and Table 5. Patients suffering from OEMR had a significantly lower 10y-pEFS of 0.32 ± 0.04 vs. 0.38 ± 0.01, *p* = 0.0204, respectively. In addition, pOS was significantly inferior for OEMR patients compared to the whole cohort—0.37 ± 0.04 vs. 0.45 ± 0.01, *p* = 0.0112, respectively (Figure 1 and Table 5b). Ten-year pEFS and pOS in non-OEMR vs. OEMR differed based on established risk stratification and were correlated with outcome (Figure 2). Patients experienced a 10-year pEFS and pOS in non-OEMR SR vs. HR of 0.51 ± 0.01 vs. 0.20 ± 0.01 and 0.59 ± 0.01 vs. 0.24 ± 0.01, *p* < 0.001, respectively, and a 10-year pEFS and pOS in OEMR SR vs. HR of 0.48 ± 0.06 vs. 0.12 ± 0.04 and 0.54 ± 0.06 vs. 0.15 ± 0.05, *p* < 0.001, respectively (Figure 2). 

**Figure 3:**
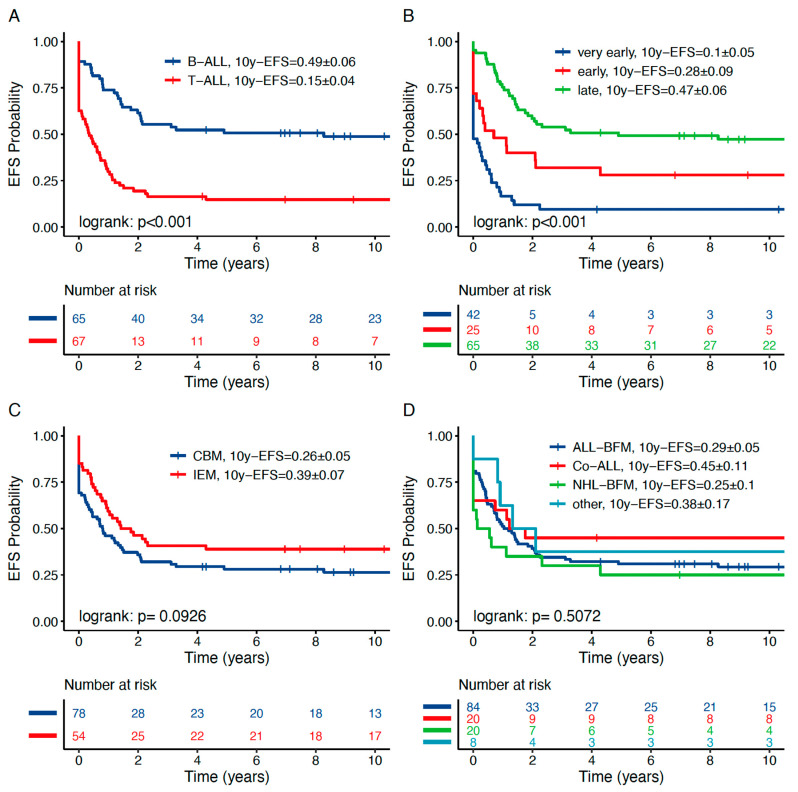
Ten-year pEFS (**A**–**D**) of OEMR patients in defined demographic subgroups. (**A**) T-ALL, (**B**) very early relapse and (**C**) combined BM relapse are correlated with significantly decreased 10-year pEFS. (**D**) Previous treatment protocol is not associated with outcome in OEMR patients. Calculation based on Kaplan–Meier analysis. *p* < 0.05.

We further focused on risk factors predicting inferior outcome within the OEMR subgroup. In that regard, immunophenotype and time to relapse were significantly associated with outcome. The 10y-pEFS and 10y-pOS of BCP-ALL OEMR patients were significantly superior to those of T-ALL OEMR patients (0.49 ± 0.06 vs. 0.15 ± 0.04 and 0.52 ± 0.06 vs. 0.22 ± 0.05, *p* < 0.001, respectively; Figure 3a; Supplementary Figure S2 and Table 5a,b). Time to first relapse confers an additional significant risk factor in the OEMR cohorts as described before for the entire relapsed ALL cohorts [36,37]. Very early OEMRs were found to have the worst prognosis compared to late OEMR: 10-year pEFS and 10-year pOS of 0.10 ± 0.05 vs. 0.47 ± 0.06, *p* < 0.001, and 0.14 ± 0.05 vs. 0.53 ± 0.06, *p* < 0.001, respectively (Figure 3b and Table 5a,b). Isolated OEMR has been associated with a superior prognosis compared to combined OEMR: 10-year pOS of 0.50 ± 0.07 vs. 0.27 ± 0.05, *p* = 0.014, respectively (Figure 3c and Table 5b). Age, previous protocol (Figure 3d) and gender do not confer an additional risk factor in the OEMR cohort.

**Figure 4:**
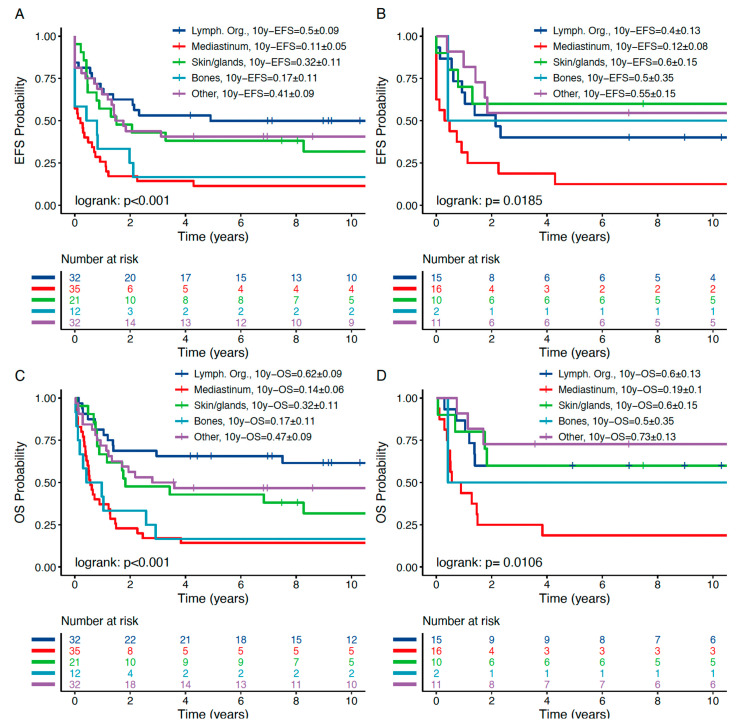
Ten-year pEFS (**A**,**B**) and pOS (**C**,**D**) of OEMR subgroups; (**A**,**C**): pEFS and pOS of combined bone marrow and isolated OEMR; (**B**,**D**): pEFS and pOS of isolated OEMR. Mediastinal and bone OEMR demonstrate inferior outcome compared to all other subgroups. Calculation based on Kaplan–Meier analysis. *p* < 0.05.

Analyzing 10y-pEFS amongst the OEMR subgroups revealed significant differences in outcomes (Figure 4 and Table 5c): “lymphatic organs”, “other” and “skin and glands” OEMR groups had comparably better 10y-pEFS of 0.50 ± 0.09, 0.41 ± 0.09 and 0.32 ± 0.11, *p* < 0.001, respectively. Mediastinal relapse was found to be associated with a very low 10y-pEFS of 0.11 ± 0.05. Consequently, mediastinal relapses most likely contributed to the lower pEFS of the entire OEMR patient cohort. The 12 patients suffering from OEMR of “bone” experienced a similarly dismal 10-year pEFS of only 0.17 ± 0.11. In contrast to “mediastinal” OEMR, “bone” OEMR comprised predominantly BCP-ALL patients relapsing very early and early (11 out of 12 patients demonstrated BCP-ALL phenotype, 92%; Table 2). Limiting the analysis to isolated OEMR, event-free survival of patients with isolated “mediastinal” relapse remained very poor (10y-pEFS 0.12 ± 0.08). On the other hand, the isolated “skin and gland” relapse group showed an excellent 10y-pEFS of 0.60 ± 0.15 (Figure 4).

**Figure 5:**
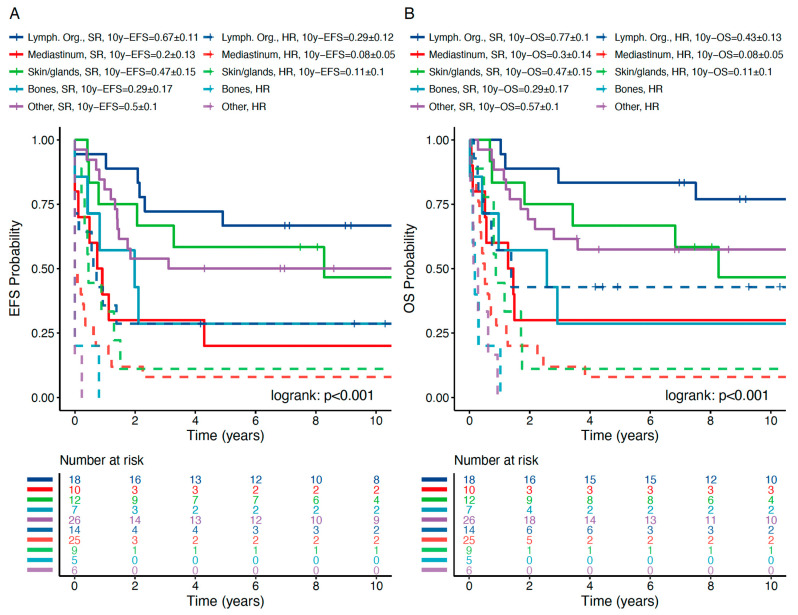
Ten-year pEFS (**A**) and pOS (**B**) of OEMR subgroups stratified by HR and SR criteria. HR OEMR is associated with significantly decreased 10-year pEFS and pOS except in mediastinal subgroup. Calculation based on Kaplan–Meier analysis. Pairwise log-rank applied in subgroup analysis. *p* < 0.05.

In addition to EFS, pOS differed significantly within the various OEMR subgroups (Figure 5 and Table 5b). Patients suffering from “mediastinal” OEMR were found to have a dismal prognosis with a pOS of only 0.14 ± 0.06 compared to patients who suffer an OEMR in “lymph nodes” who can expect a 10-year pOS of 0.62 ± 0.09. Interestingly, pOS within the isolated OEMR was excellent in the “other” group, i.e., 0.73 ± 0.13.

We further focused on outcome in OEMR SR vs. HR groups (for definitions please refer to Section 2). SR and HR stratification in OEMR revealed pEFS and pOS in the expected range in all but the mediastinal subgroup (Figure 5 and Table 5c). As in non-OEMR, T-ALL is associated with inferior outcome independent from other risk factors (Supplementary Figure S1) and should be treated according to the HR group with HSCT indication for all patients. 

In multivariate Cox regression analysis on EFS and OS, the established risk factors age, time to relapse, site of relapse and immunophenotype were revealed to be independent prognostic factors. In addition, OEMR conferred an independent risk for inferior EFS and OS (hazard ratio 1.7 and 1.7, respectively; *p* < 0.001, Table 6). Excluding T-LBL treated on a former NHL regimen from multivariate Cox regression analysis revealed an independent correlation of OEMR with EFS and OS (hazard ratio 1.66 and 1.72, respectively, *p* < 0.001). Excluding all relapsed T-ALL and T-LBL patients from the Cox regression analysis demonstrated OEMR as an independent risk factor for OS in relapsed BCP-ALL/LBL patients (hazard ratio 1.48, *p* = 0.038).

## 4. Discussion

With this report, we present retrospective data covering a period of 32 years on outcome of 132 children with extramedullary ALL relapse other than CNS or testis summarizing those enrolled into five consecutive ALL relapse trials (ALL-REZ BFM) and/or the disease-specific registries in Germany, Austria and Switzerland and single centers in the Czech Republic and Canada. OEMR is a rare event and represents only 5.7% of ALL relapses registered in that period. Patients with relapse of lymphoblastic lymphoma have been treated within trials of the ALL-REZ BFM study group and represent 15% of the OEMR group. Involved OEM localizations most often include lymph node or mediastinum as extramedullary compartment but also include a variety of rare manifestations. 

The complete OEM relapse group showed inferior prognosis compared to non-OEMR patients mainly due to poor outcome of mediastinal and bone OEMR. Whereas most patients with OEMR were adequately stratified into a risk group according to established factors such as time to relapse, immunophenotype and site of relapse, defined subgroups of patients seemingly require treatment intensification: patients with late T-ALL isolated mediastinal or lymphatic organ relapse formerly stratified into a standard risk group have poor outcome and need to receive allogeneic HSCT and possibly additional local irradiation. 

In our analysis, the subgroups “lymphatic organs” and “skin and glands” had relatively good outcomes. Both seem to be compartments where systemic chemotherapy can act without major obstacles. Considering the favorable 10y-pEFS of “skin and glands” relapse patients treated exclusively with chemotherapy, this location does not need any additional local therapy. In contrast, the subgroup “bone relapse” showed a very poor outcome. It was characterized by a high proportion of B-precursor cell immunophenotype (77%) and combined bone marrow relapses (77%). We hypothesize that particularly aggressive cells that infiltrate the surrounding bone tissue from the adjacent bone marrow might be responsible for that observation. Biopsy including deep molecular characterization, comparison to molecular features of previous lines of disease and deconvolution of clonal evolution could enable deeper insights into the biology of these very rare and aggressive relapse types. In addition, this subgroup might probably benefit from treatment intensification with irradiation and/or allogeneic HSCT. However, due to the limited number of reports on bone relapses and only 12 patients being diagnosed with bone relapses in the current analysis, explicit conclusions are cannot be driven and the indication for radiotherapy needs to be made on an individual basis [4,5].

In addition, the subgroup “other compartment” included extremely heterogeneous localizations. Thus, it was difficult to evaluate these as a single group. We tried to focus on some of these unusual locations of relapse. There were four relapses of the orbit, eyes or optic nerve. Three of these underwent radiotherapy and all of them survived in complete remission. Orbital relapses are considered sanctuary sites [38], and as reported in the past, radiotherapy might be beneficial for this site [6,7,8]. 

Based on the treating physician’s discretion, individual relapsed patients with T-LBL and pB-LBL have been included in ALL relapsed protocols. The mediastinal relapse group showed a high proportion of primary T-cell LBL and a very dismal prognosis without significant differences in 10y-pEFS between the whole group (10y-pEFS 0.11 ± 0.05) and isolated mediastinal relapses (10y-pEFS 0.12 ± 0.08). Most patients died within 10 years. According to the analysis of relapse of T-cell LBL patients treated with BFM protocols, long-term survival was only achieved in a few patients (4 of 28 patients) who were able to undergo allogeneic SCT [39]. Survival improved slightly over the last years in T-LBL patients treated on intensive relapse protocols and currently reaches 27% 8-year OS. However, cure for most patients is unattainable, and more effective treatments for T-cell LBL patients are urgently needed [40]. Intensifying induction chemotherapy and improved molecular characterization [41,42] might lead to more efficient therapies. While the therapeutic effect of mediastinal irradiation has not been confirmed in pediatric patients, some reports presented the efficacy of mediastinal irradiation for selected adult patients who responded insufficiently to induction chemotherapy [43,44]. However, a general recommendation of radiation during early induction is not feasible, since systemic therapy might be postponed, increasing the risk of systemic relapse in that rapidly proliferating disease. The current approach of the NHL BFM group includes a mediastinal boost combined with TBI in case of a detectable mediastinal mass before HSCT. Since 309 T-ALL relapses have been reported from 1983 to 2015 and mediastinal relapse is common in most T-ALL relapses (at first diagnosis up to 60% present with mediastinal mass [45]), mediastinal relapse patient numbers might be underestimated in our OEMR cohort.

It has always been a matter of debate if OEMR requires additional local consolidation including radiation. Since treatment in our cohort was triggered by local poor response or by specific localization such as mediastinal or eye/orbit, only 17 patients, the minority nonresponders, were treated with radiotherapy; thus, the impact of local irradiation on outcome cannot be determined in this retrospective analysis. 

In the ALL-REZ BFM 2002 trial, patients with isolated extramedullary relapse did not have an indication for allogeneic HSCT due to acceptable outcome for patients without bone marrow involvement [46]. Nevertheless, patients with isolated OEMR and a T-cell immunophenotype experience such a dismal outcome that chemotherapy alone is no longer an acceptable approach. Current recommendations include HSCT as definitive consolidation in very early and early isolated EM relapsed ALL patients. Evaluation of that approach is ongoing [47,48]. 

The current analysis includes patients from 1983 to 2015. The vast majority of these patients have not been deeply molecularly characterized. Treatments have been based on established chemotherapeutic schedules, irradiation and HSCT. Current immunologic approaches in relapsed/refractory treatment include daratumumab, blinatumomab, inotuzumab and CAR T-cell approaches [49,50,51,52]. Although the efficacy of these agents in the BM compartment has been clearly demonstrated, efficacy in EM localizations is less clear. Further prospective investigations will show if relapse patterns change, if EM relapse is observed more frequently and if additional consolidative elements need to be combined with immunotherapeutic approaches to prevent EM relapse and improve long-term outcome.

## 5. Conclusions

This retrospective analysis presented the outcome of ALL and LBL relapses in extramedullary compartments other than CNS or testis of which little is known so far. We were able to show that OEMR confers an independent risk for inferior pEFS and pOS and that OEMR subgroups differ significantly in regard to demographic patterns and outcome. Of high importance, we are able to show that established risk stratification can be applied to OEMR patients and these should be treated on established protocols and treatment algorithms. HSCT should be performed in all HR T-ALL relapsed patients and HR OEMR patients. Additional radiation might be of benefit in sanctuary sites, i.e., eye and bone. However, most OEMR patients do not relapse at the initial site, highlighting that the systemic disease requires systemic induction and consolidation chemotherapy. International efforts need to be established to enable robust treatment recommendations on radiation. In that regard, response assessment by positron emission tomography (PET), being of established value in adult lymphoma [47], could exert its diagnostic value even though it is not yet established in pediatric ALL and NHL patients. PET could provide additional information on the viability of the tumor and enable treating physicians to assess local response more exactly. 

Due to the scarcity of disease and high heterogeneity, international collaboration is needed to prospectively evaluate treatment, define response criteria and substantially improve outcome of pediatric OEMR ALL patients [53].

## Figures and Tables

**Table 1:** Distribution of other extramedullary relapses (OEMRs).

Site	Group	*n*	%
LN	Lymphat. organs	32	24.2
Skin	Skin/glands	14	10.6
Mediastinum/thymus	Mediastinum	35	26.5
Tonsils	Skin/glands	2	1.5
Female genital organs	Other	6	4.5
Eye/nervus opticus	Other	4	3.0
Bones	Bone	12	9.1
Paranasal sinuses/ENT	Other	2	1.5
Kidney	Other	9	6.8
Liver	Other	3	2.3
Pancreas	Other	1	0.8
Serosae (pleural/cardial/joints)	Skin/glands	1	0.8
Glands (mammae/g. parotis/g. lacrimae)	Skin/glands	4	3.0
Spleen	Other	1	0.8
Colon/intestine	Other	1	0.8
Epidural	Other	1	0.8
Abdomen	Other	2	1.5
Other	Other	2	1.5
**Total**		132	100.0

Legend to Table 1: ENT, ear nose throat; LN, lymph node; OEM, other extramedullary.

**Table 2:** Patient characteristics.

	ALL Relapse Trial Patients		Other Extramedullary Relapse Patients					Other Extramedullary Relapse Subgroups										
			No		Yes			Lymph. Organs		Mediast. Organs		Other Compartment		Skin/Glands		Bone		
	*n*	%	*n*	%	*n*	%	*p* **	*n*	%	*n*	%	*n*	%	*n*	%	*n*	%	*p* **
**Total group**	2323	100	2191	100	132	100		32	24.2	35	26.6	32	24.2	21	15.9	12	9.1	
**Patient characteristics**																		
**Sex**							0.37											0.71
Male	1474	63	1395	63.7	79	59.8		19	59.4	24	68.6	19	59.4	11	52.4	6	50	
Female	849	37	796	36.3	53	40.2		13	40.6	11	31.4	13	40.6	10	47.6	6	50	
**Time point of relapse**							0.01											0.04
Very early	565	24.3	523	23.9	42	31.9		11	34.4	15	42.9	5	15.6	6	28.6	5	41.7	
Early	691	29.8	666	30.4	25	18.9		5	15.6	11	31.4	5	15.6	2	7.1	2	16.6	
Late	1067	45.9	1002	45.7	65	49.2		16	50	9	25.7	22	68.8	13	46.4	5	41.7	
**Age at relapse**							0.38											0.25
≤5 years	386	16.6	369	16.8	17	12.9		4	12.5	5	14.3	2	6.3	2	9.5	4	33.3	
≥5 years and ≤10 years	1011	43.5	955	43.7	56	42.4		14	43.8	17	48.5	17	53.1	6	28.6	2	16.7	
>10 years and ≤15 years	648	27.9	610	27.8	38	28.8		9	28.1	10	28.6	9	28.1	6	28.6	4	33.3	
>15 years and <20 years	278	12.0	257	11.7	21	15.9		5	15.6	3	8.6	4	12.5	7	33.3	2	16.7	
**Site of relapse**							**<0.001**											0.32
Isolated BM	1439	62.0	1439	65.7	0	0												
Combined BM and EM	505	21.7	427	19.5	78	59.1		17	53.1	19	54.3	21	65.6	11	52.4	10	83.3	
Isolated extramedullary	379	16.3	325	14.8	54	40.9		15	46.9	16	45.7	11	34.4	10	47.6	2	16.7	
**Immunophenotype**							**<0.001**											**<0.001**
Precursor B cell	2014	86.7	1949	89	65	49.2		14	43.8	2	5.7	23	71.9	15	71.4	11	91.7	
T cell	309	13.3	242	11	67	50.8		18	56.2	33	94.3	9	28.1	6	28.6	1	8.3	
**Therapy**							0.29											0.23
Chemotherapy/radiotherapy exclusively	1550	66.7	1459	66.7	91	68.9		18	56.2	26	74.3	23	71.9	14	66.7	10	83.4	
Allogeneic SCT	664	28.6	632	28.8	32	24.2		12	37.5	7	20	5	15.6	7	33.3	1	8.3	
Autologous SCT	57	2.5	51	2.3	6	4.6		0	0	2	5.7	3	9.4	0	0	1	8.3	
No data	52	2.2	49	2.2	3	2.3		2	6.3	0	0	1	3.1	0	0	0	0	
**NHL Therapy**							**<0.001**											0.08
Other	2247	96.7	2135	97.4	112	84.8		25	78.1	26	74.3	30	93.8	20	95.2	11	91.7	
NHL-BFM	62	2.7	42	1.9	20	15.2		7	21.9	9	25.7	2	6.2	1	4.8	1	8.3	
No data	14	0.06	14	0.7	0	0												

Legend to Table 2: ** Pearson/chi-squared or Fisher’s exact test, missing values excluded. Abbreviations: BCP, B-cell precursor; BM, bone marrow; EM, extramedullary; NHL-BFM, Non-Hodgkin’s Lymphoma Berlin–Frankfurt–Munster protocol; SCT, stem cell transplantation; SE, standard error.

**Table 3:** Events within the OEMR group.

(a) All Events																												
	OEM									OEM Group																		
	No		Yes							Lymphat. Organs			Skin/Glands					Mediastinum			Bone			Other				
	*n*	%	*n*		%			*p* *		*n*	%		*n*		%			*n*		%	*n*	%		*n*	%			*p* *
**Total**	2191	100.0	132		100.0			0.036		32	100.0		21		100.0			35		100.0	12	100.0		32	100.0			0.025
**Event**	821	37.5	42		31.8			0.23		16	50.0		7		33.3			4		11.4	2	16.7		13	40.6			
**in CCR**																												
**Died in CR**	138	6.3	7		5.3			0.78		1	3.1		2		9.5			3		8.6	1	8.3		.	.			
**2nd malignoma**	29	1.3	3		2.3			0.60		1	3.1		.		.			1		2.9	1	8.3		.	.			
**Another relapse**	889	40.6	48		36.4			0.39		9	28.1		11		52.4			12		34.3	3	25.0		13	40.6			
**Nonresponder/progr. disease**	215	9.8	21		15.9			0.03		4	12.5		.		.			11		31.4	2	16.7		4	12.5			
**Death in induction**	81	3.7	11		8.3			0.02		1	3.1		1		4.8			4		11.4	3	25.0		2	6.3			
**Death of unknown origin**	4	0.2	0		0			1		.	.		.		.			.		.	.	.		.	.			
**(b) OEM Subsequent Relapse Sites**																												
	**OEM**										**OEM Group**																	
	**No**			**Yes**							**Lymphat. Organs**			**Skin/Glands**					**Mediastinum**		**Bone**				**Other**			
	***n***	**%**		***n***		**%**			*p* *		***n***	**%**		***n***			**%**		***n***	**%**	***n***		**%**		***n***		**%**	
**Subs. relapse**	889	100.0		48		100.0					9	100.0		11			100.0		12	100.0	3		100.0		13		100.0	
**Subseq_site**									<0.001																			
**IBM**	680	76.5		20		41.7					4	44.4		5			45.5		6	50.0	1		33.3		4		30.8	
**CBM**	94	10.6		8		16.7					1	11.1		2			18.2		2	16.7	2		66.7		1		7.7	
**IEM**	115	12.9		20		41.7					4	44.4		4			36.4		4	33.3	.		.		8		61.5	
**(c) Subsequent Relapse Sites Compared to OEMR First Relapse Sites**																												
							**Total**				**Subsequent OEMR**					**Subsequent Relapse CNS/Testis Only**					**Subsequent Relapse Site**					***n***		
**OEM**	**OEM group**						32																					
**LN**	**Lymphat. organs**										5					0					**LN**					4		
																					**Mediastinum**					1		
**Skin**	**Skin/glands**						14				3					3					**LN**					1		
																					**glands**					1		
																					**skin**					1		
																					**CNS/testis**					3		
**Mediastinum/thymus**	**Mediastinum**						35				3					3					**LN**					3		
																					**CNS/testis**					3		
**Tonsils**	**Skin/glands**						2				0					0										0		
**Female genital organs**	**Other**						6				0					1					**CNS/testis**					1		
**Eye/nervus opticus**	**Other**						4				0					0										0		
**Bones**	**Bone**						12				1					1					**Bones**					1		
																					**CNS/testis**					1		
**Paranasal sinuses/ENT**	**Other**						2				0					1					**CNS/testis**					1		
**Kidney**	**Other**						9				1					1					**Paranasal sinus**					1		
																					**CNS/testis**					1		
**Liver**	**Other**						3				1					0					**Liver**					1		
**Pancreas**	**Other**						1				1					0					**Pancreas**					1		
**Serosae (pleural/cardial/joints)**	**Skin/glands**						1				0					0										0		
**Glands (mammae/g. parotis/g. lacrimae)**	**Skin/glands**						4				0					0										0		
**Spleen**	**Other**						1				0					0										0		
**Colon/intestine**	**Other**						1				1					0					**Skin**					1		
**Epidural**	**Other**						1				1					0					**Kidney**					1		
**Abdomen**	**Other**						2				0					0										0		
**Other**	**Other**						2				1					0					**Other**					1		
**Total**							132				18					10										28		

Legend to Table 3: * Pearson/chi-squared including Yate’s continuity correction; due to the exploratory character no correction for multiple testing has been performed. Missing values excluded. Abbreviations: BM, bone marrow; (C)CR, (continued) complete remission; IBM, isolated bone marrow; CBM, combined bone marrow; IEM, isolated extramedullary; LN, lymph node; CNS, central nervous system; OEM, other extramedullary; w/o, without.

**Table 4:** Events following HSCT.

(a) HSCT Performed Per Risk Group in OEMR and Non-OEMR Patients																														
		HSCT—OEMR																												
		Total					S1								S2				S4											
		*n*			%		*n*			%					*n*		%		*n*			%								
Total *		132			100		30			100					59		100		43			100								
**No HSCT**		91			68.9		20			66.6					39		66.1		32			74.5								
**Allogeneic HSCT**		32			24.2		5			16.7					18		30.5		9			20.9								
**Autologous HSCT**		6			4.5		3			10					2		3.4		1			2.3								
**Unknown**		3			2.4		2			6.7									1			2.3								
		**HSCT—Non-OEMR**																												
		**Total**					**S1**					**S2**					**S3**					**S4**								
		***n***			**%**		***n***			**%**		***n***			**%**		***n***		**%**			***n***		**%**						
Total *		2190			100		71			100		1299			100		320		100			500		100						
**No HSCT**		1459			66.7		67			94.3		915			70.4		162		50.6			315		63.0						
**Allogeneic HSCT**		631			28.8		0			0		326			25.1		145		45.3			160		32.0						
**Autologous HSCT**		51			2.3		1			1.4		26			2		9		2.8			15		3.0						
**Unknown**		49			2.2		3			4.2		32			2.5		4		1.3			10		2.0						
**(b) HSCT, All Events in Non-OEMR and OEMR Groups**																														
	**HSCT—Events**																													
	**Total** **Non-OEMR**			**Total** **OEMR**				**No HSCT** **Non-OEMR**			**No HSCT OEMR**			**Allogeneic HSCT Non-OEMR**				**Allogeneic HSCT OEMR**			**Autologous** **Non-OEMR**				**Autologous** **OEMR**		**Unknown** **Non-OEMR**		**Unknown** **OEMR**	
	***n***		**%**	***n***		**%**		***n***	**%**		***n***		**%**	***n***		**%**					***n***		**%**						***n***	***%***
Total **	1877		100	100		100		1150	100		60		100	628		100		32		100	51		100		6	100	48	100	2	100.0
**Event**	821		43.7	42		42.0		426	37		23		38.3	337		53.6		14		43.8	13		25.5		3	50	45	93.8	2	100.0
**in CCR**																														
**Died in CR**	138		7.3	7		7.0		41	3.6		3		5.0	96		15.3		4		12.5	1		2							
**2nd malignoma**	29		1.5	3		3.0		13	1.1		1		1,7	15		2.4		2		6.3	1		2							
**Subsequent relapse**	889		47.3	48		48.0		670	58.3		33		55.0	180		28.7		12		37.5	36		70.5		3	50	3	6.2		

Legend to Table 4: * One patient excluded due to unknown risk group. ** Progressive disease, death in induction and death unknown excluded. Abbreviations: (C)CR, (continued) complete remission; HSCT, hematopoietic stem cell transplantation; OEM(R), other extramedullary (relapse); w/o, without.

**Table 5:** pEFS and pOS in non-OEMR, all OEMR and OEMR subgroups.

(a) pEFS					
		OEMR			
		No—2191		Yes—132	
		pEFS ± SE (10 Years)	*p* *	pEFS ± SE (10 Years)	*p* *
**Total group**		0.38 ± 0.01		0.32 ± 0.04	0.0204
**Patient characteristics**					
** ** **Sex**			0.49		0.18
Male		0.38 ± 0.01		0.28 ± 0.05	
Female		0.39 ± 0.02		0.38 ± 0.07	
** ** **Time point of relapse**			<0.001		<0.001
Very early		0.20 ± 0.02		0.10 ± 0.05	
Early		0.29 ± 0.02		0.28 ± 0.09	
Late		0.54 ± 0.02		0.47 ± 0.06	
** ** **Age at relapse**			<0.001		0.60
≤5 years		0.31 ± 0.02		0.29 ± 0.11	
≥5 years and ≤10 years		0.42 ± 0.02		0.37 ± 0.07	
>10 years and ≤15 years		0.37 ± 0.02		0.24 ± 0.07	
>15 years and <20 years		0.38 ± 0.03		0.33 ± 0.10	
** ** **Site of relapse**			<0.001		0.093
Isolated BM		0.34 ± 0.01		--	
Combined BM and EM		0.45 ± 0.02		0.26 ± 0.05	
Isolated extramedullary		0.49 ± 0.03		0.39 ± 0.07	
** ** **Immunophenotype**			<0.001		<0.001
Precursor B cell		0.40 ± 0.01		0.49 ± 0.06	
T cell		0.20 ± 0.03		0.15 ± 0.04	
** ** **Therapy**			<0.001		0.010
Chemotherapy/radiotherapy only		0.30 ± 0.01			
				0.25 ± 0.05	
Allogeneic SCT		0.54 ± 0.02		0.42 ± 0.09	
Autologous SCT		0.25 ± 0.06		0.50 ± 0.20	
No data		0.92 ± 0.04		0.67 ± 27	
** ** **NHL Therapy**			0.0043		0.76
Other		0.38 ± 0.01		0.33 ± 0.04	
NHL-BFM		0.26 ± 0.07		0.25 ± 0.10	
**(b) pOS**					
		**OEMR**			
		**No—2191**		**Yes—132**	
		**pOS ± SE (10 years)**	*p* *	**pOS ± SE (10 years)**	*p* *
**Total group**		0.45 ± 0.01		0.37 ± 0.04	0.0112
**Patient characteristics**					
**Sex**			0.888		0.114
Male		0.45 ± 0.01		0.31 ± 0.0	
Female		0.44 ± 0.02		0.45 ± 0.02	
**Time point of relapse**			<0.001		<.001
Very early		0.23 ± 0.02		0.14 ± 0.05	
Early		0.34 ± 0.02		0.32 ± 0.09	
Late		0.63 ± 0.02		0.53 ± 0.06	
**Age at relapse**			<0.001		0.656
≤5 years		0.37 ± 0.03		0.35 ± 0.12	
≥5 years and ≤10 years		0.50 ± 0.02		0.42 ± 0.07	
>10 years and ≤15 years		0.43 ± 0.02		0.29 ± 0.07	
>15 years and <20 years		0.41 ± 0.03		0.37 ± 0.11	
**Site of relapse**			<0.001		0.014
Isolated BM		0.41 ± 0.01		--	
Combined BM and EM		0.49 ± 0.02		0.27 ± 0.05	
Isolated extramedullary		0.56 ± 0.03		0.50 ± 0.07	
**Immunophenotype**			<0.001		<0.001
Precursor B cell		0.47 ± 0.01		0.52 ± 0.06	
T cell		0.23 ± 0.03		0.22 ± 0.05	
**Therapy**			<.001		0.0055
Chemotherapy/radiotherapy exclusively		0.38 ± 0.01			
				0.30 ± 0.05	
Allogeneic SCT		0.59 ± 0.02		0.47 ± 0.09	
Autologous SCT		0.33 ± 0.07		0.50 ± 0.20	
No data		096 ± 0.03		0.67 ± 0.27	
**NHL Therapy**			0.0069		0.7645
Other		0.45 ± 0.01		0.36 ± 0.05	
NHL-BFM		0.31 ± 0.07		0.40 ± 0.11	
**(c) pEFS and pOS in OEMR Subgroups**					
	**OEMR Subgroups**				
	***n***	**pEFS ± SE (10 years)**	*p* *	**pOS ± SE (10 years)**	*p* *
			**<0.001**		**<0.001**
**Lymph. organs**	32	0.50 ± 0.09		0.62 ± 0.09	
**SR**	18	0.67 ± 0.11	**0.005**	0.77 ± 0.10	**0.015**
**HR**	14	0.29 ± 12		0.43 ± 0.13	
**Mediast. organs**	35	0.11 ± 0.05		0.14 ± 0.06	
**SR**	10	0.20 ± 0.13	**0.113**	0.30 ± 0.14	**0.117**
**HR**	25	0.08 ± 0.05		0.08 ± 0.05	
**Other compartment**	32	0.41 ± 0.09		0.47 ± 0.09	
**SR**	26	0.50 ± 0.10	**<0.001**	0.57 ± 0.10	**<0.001**
**HR**	6	**		**	
**Skin/glands**	21	0.32 ± 0.11		0.32 ± 0.11	
**SR**	12	0.47 ± 0.15	**0.010**	0.47 ± 0.15	**0.01**
**HR**	9	0.11 ± 0.10		0.11 ± 0.10	
**Bone**	12	0.17 ± 0.11		0.17 ± 0.11	
**SR**	7	0.29 ± 0.17	**0.01**	0.29 ± 0.17	**0.035**
**HR**	5	**		**	

Legend to Table 5: * Log-rank test and pairwise log-rank test, missing values excluded. ** Ten-year pEFS and pOS not reached. Abbreviations: BM, bone marrow; EM, extramedullary; HR, high risk; NHL-BFM, Non-Hodgkin’s Lymphoma Berlin–Frankfurt–Munster protocol; OEM(R), other extramedullary (relapse); pEFS, probability of event-free survival; pOS probability of overall survival; SCT, stem cell transplantation; SE, standard error; SR, standard risk.

**Table 6:** Cox regression; multivariate analysis.

(a) EFS; Cox Regression; Multivariate Analysis						
	Univariate Analysis			Mulitvariate Analysis		
	HR	95% CI	*p* (chi)	HR	95% CI	*p* (chi)
**Gender: ref. male**						
female	0.95	0.85–1.06	0.33			
**Age: ref. < 5 years**						
age > 5 to ≤ 10 years	0.7	0.6–0.8	0.009	1.13	0.97–1.31	0.13
age > 10 to ≤ 15 years	0.81	0.7–0.95	0.03	1.33	1.13–1.56	<0.001
age > 15 years	0.81	0.67–0.98	0.017	1.24	1.02–1.52	0.03
**OEM: ref. no OEMR**						
OEMR	1.30	1.05–1.06	<0.001	1.76	1.39–2.23	<0.001
**Time: ref. very early**						
early	0.57	0.5–0.64	<0.001	0.65	0.57–0.075	<0.001
late	0.26	0.23–0.29	<0.001	0.24	0.21–0.028	<0.001
**Site: ref. IBM**						
CBM	0.81	0.71–0.92	<0.001	0.68	.059–0.78	<0.001
IEM	0.67	0.57–0.78	<0.001	0.40	0.34–0.47	<0.001
**Phenotype: ref. BCP**						
T-ALL	2.37	2.06–2.72	<0.001	1.61	1.38–1.88	<0.001
**NHL protocol: ref. no**						
NHL-BFM	1.68	1.25–2.23	<0.001	1.25	0.92–1.70	0.80
**(b) OS; Cox Regression; Multivariate Analysis**						
	**Univariable Analysis**			**Mulitvariable Analysis**		
	**HR**	**95% CI**	***p* (chi)**	**HR**	**95% CI**	***p* (chi)**
**Gender: ref. male**						
female	0.99	0.88–1.11	0.821			
**Age: ref. < 5 years**						
age > 5 to ≤ 10 years	0.66	0.57–0.77	<0.001	1.08	0.92–1.27	0.33
age > 10 to ≤ 15 years	0.82	0.69–0.96	0.013	1.33	1.12–1.57	<0.001
age > 15 years	0.88	0.72–1.07	0.205	1.41	1.15–1.73	<0.001
**OEM: ref. no OEMR**						
OEMR	1.33	1.07–1.66	0.011	1.71	1.33–1.29	<0.001
**Time: ref. very early**						
early	0.57	0.5–0.65	<0.001	0.65	0.57–0.75	<0.001
late	0.24	0.21–0.27	<0.001	0.22	0.19–0.75	<0.001
**Site: ref. IBM**						
CBM	0.87	0.76–0.99	0.042	0.72	0.62–0.84	<0.001
IEM	0.63	0.54–0.75	<0.001	0.38	0.32–0.45	<0.001
**Phenotype: ref. BCP**						
T-ALL	2.41	2.09–2.78	<0.001	1.62	1.38–1.89	<0.001
**NHL protocol: ref. no**						
NHL-BFM	1.56	1.14–2.13	0.005	1.21	0.88–1.70	0.82

Legend to Table 6: Abbreviations: BCP, B-cell precursor; chi, chi-squared test; CBM, combined bone marrow relapse; CI, confidence interval; HR, hazard ratio; IEM, isolated bone marrow relapse; NHL-BFM, Non-Hodgkin’s Lymphoma Berlin–Frankfurt–Munster protocol; OEMR, other extramedullary relapse.

## Supplementary Materials

The following are available online at https://www.mdpi.com/article/10.3390/jcm10225292/s1, Figure S1: Distribution of OEMR, Figure S2: T-ALL relapses, Table S1: Genetic characteristics, Table S2: Radiation in non-OEMR and OEMR patients.
