# Supplementary material for: Other (Non-CNS/Testicular) Extramedullary Localizations of Childhood Relapsed Acute Lymphoblastic Leukemia and Lymphoblastic Lymphoma—A Report from the ALL-REZ Study Group
Source: J Clin Med. 2021 Nov 14;10(22):5292. doi: 10.3390/jcm10225292 (PMC8621955; doi:10.3390/jcm10225292)
Supplement: Supplementary file 1 [file jcm-10-05292-s001.zip › jcm-1439420-supplementary.pdf]

Supplementary

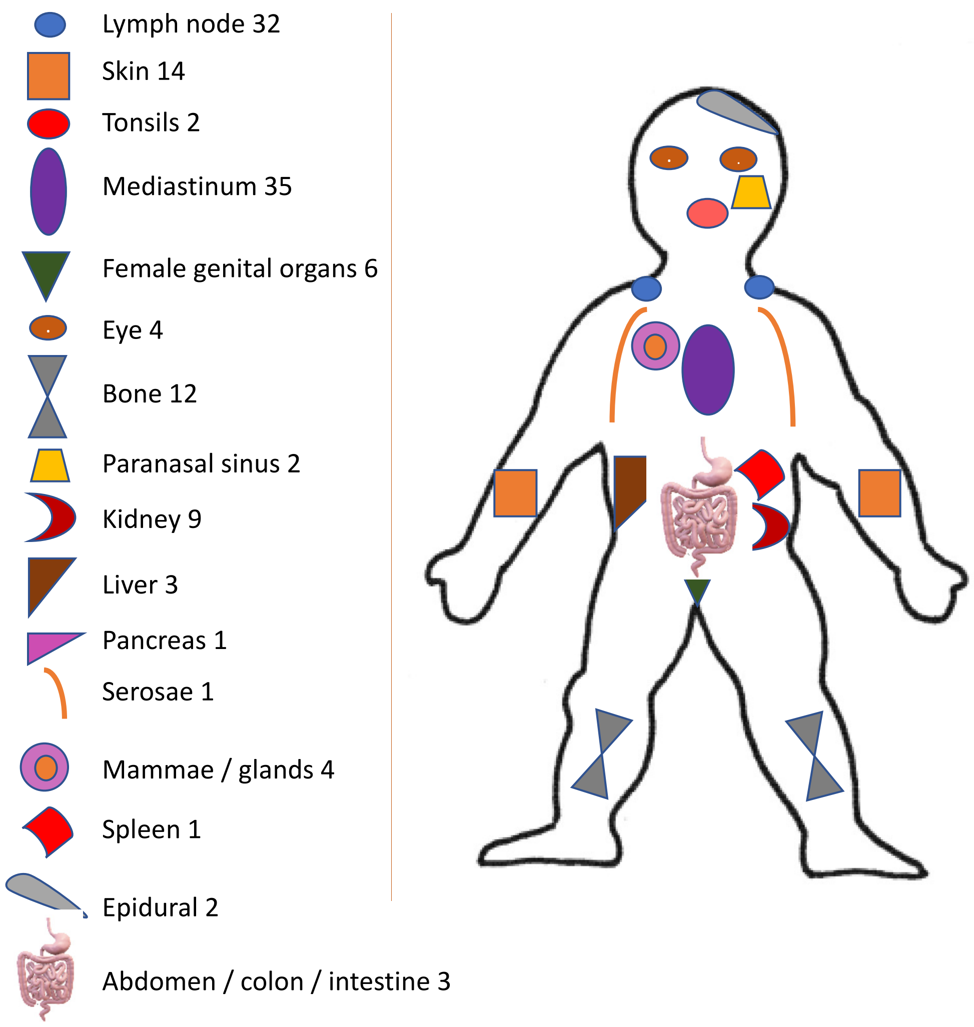

Figure S1: Distribution of other extramedullary ALL relapses.

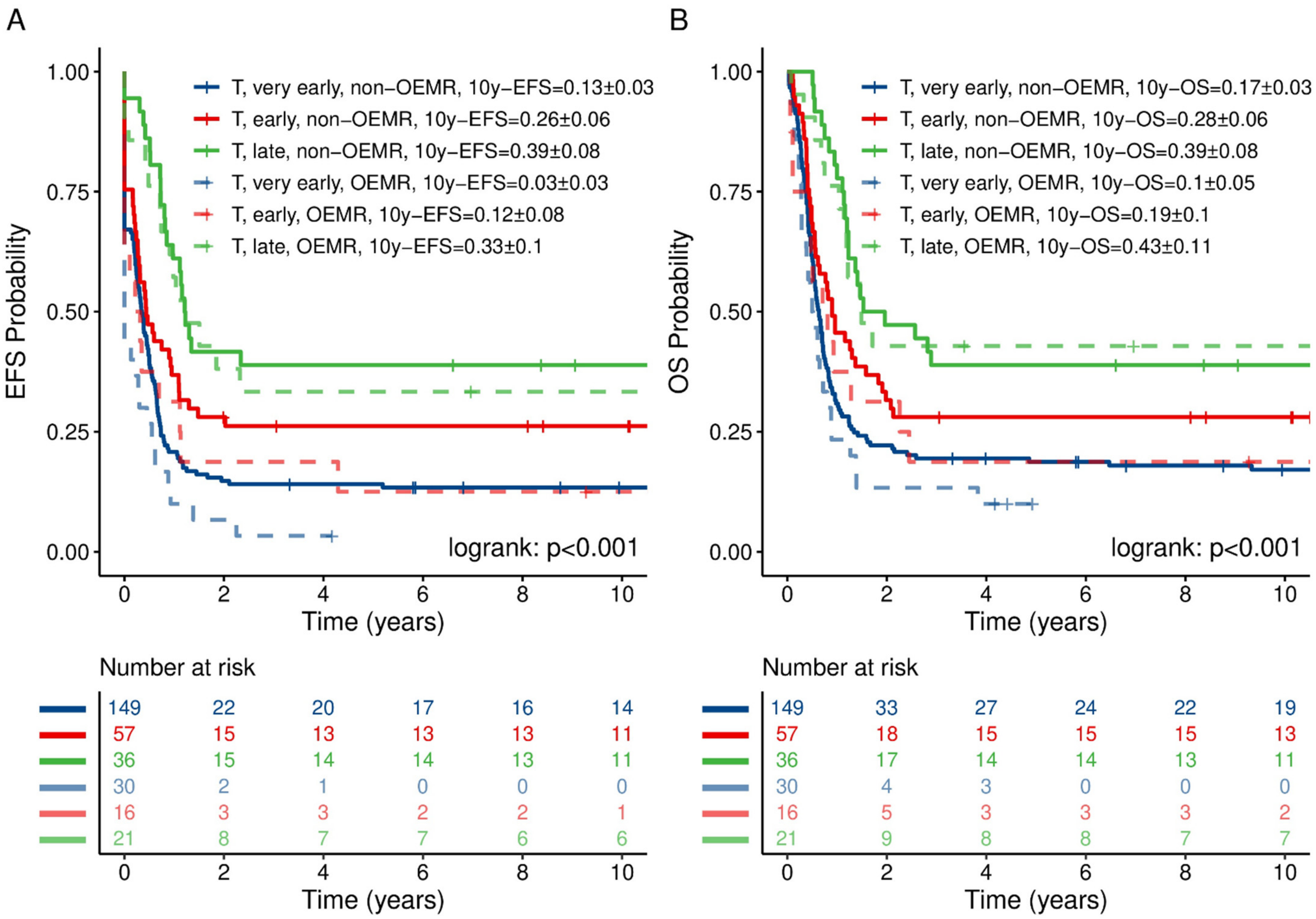

Figure S2. T-ALL relapses. pEFS (A) and pOS (B) of T-ALL OEMR and T-ALL non-OEMR depending on time to relapse.

Suppl. Table S1 genetic characteristics.

	OEM								OEMgroup									
	Total		no		yes		.		Lymphat. organs		Skin/glands		Mediastinum		Bone		Other	
	N	%	N	%	N	%	N	%	N	%	N	%	N	%	N	%	N	%
**Total**	2323	100.0	2191	100.0	132	100.0	2191	100.0	32	100.0	21	100.0	35	100.0	12	100.0	32	100.0
**genexp**																		
**negative**	651	28.0	610	27.8	41	31.1	610	27.8	12	37.5	9	42.9	8	22.9	1	8.3	11	34.4
**BCR-ABL+**	20	0.9	19	0.9	1	0.8	19	0.9	.	.	1	4.8	.	.	.	.	.	.
**ETV6-RUNX1+**	132	5.7	131	6.0	1	0.8	131	6.0	.	.	.	.	.	.	.	.	1	3.1
**MLL-AF4+**	13	0.6	13	0.6	.	.	13	0.6	.	.	.	.	.	.	.	.	.	.
**not done**	711	30.6	663	30.3	48	36.4	663	30.3	11	34.4	7	33.3	17	48.6	5	41.7	8	25.0
**not reported**	796	34.3	755	34.5	41	31.1	755	34.5	9	28.1	4	19.0	10	28.6	6	50.0	12	37.5

Legend to Suppl table 1: Abbreviations: BCR-ABL, Abelson-Tyrosinkinase (Abl)-Breakpoint Cluster Region; MLL-AF4, myeloid/lymphoid or mixed-lineage leukemia- ALL1 fused gene on Chromosome 4; ETV6-RUNX1, Ets-leukemia virus- Runt-related transcription factor 1; OEM, other extramedullary.

Suppl Table S2 Radiation in non OEMR and OEMR patients

	OEMR		OEMgroup									
	total		Lymphat. organs		Skin/glands		Mediastinum		Bone		Other	
	**N**	**%**	**N**	**%**	**N**	**%**	**N**	**%**	**N**	**%**	**N**	**%**
**Total**	132	100.0	32	100.0	21	100.0	35	100.0	12	100.0	32	100.0
**radiation**												
no	82	62.1	20	62.5	14	66.7	17	48.6	10	83.3	21	65.6
yes	46	34.9	12	37.5	7	33.3	16	45.7	1	8.3	10	31.2
unknown	4	3.0	.	.	.	.	2	5.7	1	8.3	1	3.1
**local radiation or TBI or TBI+boost**												
local	15	11.4	.	.	2	9.5	9	25.7	.	.	4	12.5
TBI	29	22.0	12	37.5	5	23.8	6	17.1	1	8.3	5	15.6
TBI + local boost	2	1.5	.	.	.	.	1	2.9	.	.	1	3.1
unknown	4	3.0	.	.	.	.	2	5.7	1	8.3	1	3.1
